# Protein Targeting to Starch 1 is essential for starchy endosperm development in barley

**DOI:** 10.1093/jxb/ery398

**Published:** 2018-11-08

**Authors:** Yingxin Zhong, Andreas Blennow, Olivia Kofoed-Enevoldsen, Dong Jiang, Kim Henrik Hebelstrup

**Affiliations:** 1 Department of Molecular Biology and Genetics, Aarhus University, Flakkebjerg, Forsøgsvej, Slagelse, Denmark; 2 Department of Plant and Environmental Sciences, Copenhagen University, Frederiksberg, Denmark; 3 National Technique Innovation Center for Regional Wheat Production/Key Laboratory of Crop Physiology and Ecology in Southern China, Ministry of Agriculture/National Engineering and technology Center for Information Agriculture, Nanjing Agricultural University, Nanjing, PR China

**Keywords:** Barley, cereal grain, CRISPR/Cas9, GBSS, PTST1, starch biosynthesis, *waxy*

## Abstract

Plant starch is the main energy contributor to the human diet. Its biosynthesis is catalyzed and regulated by co-ordinated actions of several enzymes. Recently, a factor termed Protein Targeting to Starch 1 (PTST1) was identified as being required for correct granule-bound starch synthase (GBSS) localization and demonstrated to be crucial for amylose synthesis in Arabidopsis. However, the function of its homologous protein in storage tissues (e.g. endosperm) is unknown. We identified a PTST1 homolog in barley and it was found to contain a crucial coiled-coil domain and carbohydrate-binding module. We demonstrated the interaction between PTST1 and GBSS1 by fluorescence resonance energy transfer (FRET) in barley endosperm. By tagging PTST1 with the fluorophore mCherry, we observed that it is localized in the stroma of barley endosperm amyloplasts. PTST1 overexpression in endosperm increased endogenous *gbss1a* gene expression and amylose content. *Gbss1a* and *ptst1* mutants were generated using clustered regularly interspaced short palindromic repeats (CRISPR)/CRISPR-related protein 9 (Cas9)-based targeted mutagenesis. Homozygous *gbss1a* mutants showed a waxy phenotype. Grains of *ptst1* mutants did not accumulate any starch. These grains dried out during the desiccation stage and were unable to germinate, suggesting that PTST1 is essential for development of starchy endosperm and viable grains.

## Teaser

Protein Targeting to Starch 1 is essential for development of starchy endosperm and viability of barley grains.

## Introduction

Starch biosynthesis in plants requires the co-ordinated actions of several enzymes, including ADP-glucose pyrophosphorylase (AGPase), granule-bound starch synthase (GBSS), soluble starch synthase (SSS), starch branching enzyme, starch debranching enzyme, glucan water dikinase, and starch phosphorylase (Blennow *et al.*, 2013). After converting glucose-1-phosphate to activated glucosyl donor ADP-glucose by AGPase, amylose and amylopectin are produced by the involvement of GBSS and SSS, respectively. In cereal endosperm, various isoforms of SS have been identified: GBSS, SSI, SSII, SSIII, and SSIV (Nakamura, 2002; James *et al.*, 2003). SSI, SSII, and SSIII elongate short, medium, and long chains of amylopectin, respectively (Nakamura *et al.*, 2005; Fujita *et al.*, 2006, 2007), while SSIV seems to be involved in initiation of starch granules (Toyosawa *et al.*, 2016). Of the two currently identified GBSS isoforms, GBSSII functions in non-storage plant tissues such as leaf, whereas the role of GBSSI is mostly confined to storage tissues such as the seed endosperm (Dian *et al.*, 2003, Hirose and Terao, 2004).

Based on metabolic diurnal dynamics, two types of starch can be identified. Transient starch is synthesized mainly in leaves during the day and degraded at night to provide carbon for non-photosynthetic metabolism. Storage starch is accumulated in storage tissue including developing seeds and tubers, and serves as a long-term carbon store mainly for germination. Due to their different physiological functions, the mechanisms for starch biosynthesis in storage and transient tissues are significantly different. For example, in Arabidopsis, Protein Targeting to Starch 2 (PTST2) mediates interaction between SSIV and MOS for initiation of transient leaf starch granules (Seung *et al.*, 2017). *Ptst2* mutants contain none or a single large starch granule in chloroplasts, while the wild type typically contains 5–7 granules. In rice endosperm, a homolog of PTST2 was identified as FLO6 (Peng *et al.*, 2014), which plays a different role in starch biosynthesis from that in Arabidopsis. FLO6 interacts with ISA1, and the *flo6* mutants produce three types of abnormal amyloplasts, each with either no starch granules or large numbers of tiny granules and abolished compound structures. Even for storage tissues in cereals, the homologous genes involved in starch biosynthesis in different species may function in different ways. The same homologous mutant of *ptst2* in barley, identified as *fra*, contains fractured starch granules rather than an altered number of starch granules (Saito *et al.*, 2018). Therefore, it is important to characterize the function of PTST homologs in different cereals and tissues.

GBSSI is encoded by the *waxy* (*wx*) locus. Mutants carrying defects in the GBSSI/WAXY gene have been isolated in many species, including wheat (Nakamura *et al.*, 1995), maize (Shure *et al.*, 1983), rice (Wang *et al.*, 1995), and potato (Hovenkamp-Hermelink *et al.*, 1987), all of which produce amylose-free starch granules. Waxy cereals offer a valuable starch quality for foods without seriously jeopardizing total starch contents or grain yield. For example, *indica* waxy rice is used in rice tamale and rice pudding for its tenderness, while *japonica* waxy rice is used for sweetened rice cakes due to its stickiness (Villareal *et al.*, 1993). Biscuits baked with partially waxy wheat flour show higher sensory scores than those with normal flour (Zhong *et al.*, 2017). Furthermore, waxy starches are used in paper manufacturing, where clear, consistent gels are required (Jobling, 2004).

GBSS-type enzymes represent the majority of the protein fraction bound to storage starch granules (Wang *et al.*, 2013). However, they do not contain known types of starch-binding domains nor have they been shown to form complexes with other soluble starch synthases or starch branching enzymes containing carbohydrate-binding domains (CBMs) (Tetlow *et al.*, 2008; Liu *et al.*, 2012). HvGBSSIa from the barley cultivar CDC Alamo showed deficient targeting to starch granules. This deficient targeting results in a waxy phenotype even though GBSS enzymes are expressed normally (Hebelstrup *et al.*, 2017). In Arabidopsis, targeting of GBSS to transient leaf starch granules has been shown to be dependent on interaction with PTST, which is a 26 kDa protein containing a glucan-binding module family 48, CBM48 (Seung *et al.*, 2015). PTST genes are highly conserved, and homologous genes exist in cereal genomes. However, it is not known whether PTST1 interacts with GBSS1a in cereals, or if it has a function in storage starch biosynthesis.

We studied the function of PTST1 in starch biosynthesis in barley endosperm. The interaction of PTST1 and GBSS1a in endosperm was confirmed by FRET (fluorescence resonance energy transfer). PTST1 overexpressors showed higher gene expression of both *ptst1* and *gbss1a* mRNA in endosperm at different development stages and they had an increased content of amylose. We also generated *gbss1a* and *ptst1* loss-of-function mutant plants and grains by clustered regularly interspaced short palindromic repeats (CRISPR)/CRISPR-related protein 9 (Cas9). The endosperms of *gbss1a* mutants were amylose-free, while *ptst1* mutant endosperms contained increased sugar levels and had no starch accumulation. Homozygous *ptst1* mutant grains did not survive desiccation and could therefore not generate progeny. Our results suggest that PTST1 is not only involved in amylose biosynthesis by interacting with GBSS1a, as in Arabidopsis, but is generally essential for development of starchy endosperm in barley grains.

## Materials and methods

### Molecular phylogenetic analyses

The amino acid sequence of AtPTST1 was used to search different proteomic databases including National Center for Biotechnology Information (http://www.ncbi.nlm.nih.gov/BLAST/) and Ensemblplants databases (http://plants.ensembl.org/; for barley and wheat). The PTST1 protein family with highest sequence similarity to AtPTST1 from different plant species was used for homology comparison using DNAMAN8 (Lynnon Biosoft). Predicted amino acid sequences of AtPTST1 homologs from different plant species are shown in Supplementary Fig. S1 at *JXB* online. Coiled-coil domain prediction was carried out by both UniProt (https://www.uniprot.org/) and the COILS/PCOILS server (https://embnet.vital-it.ch/).

### Engineering of vectors and transformation of barley

The plasmid vector pUCE-UBI:PTST1-mCherry:NOS was generated by In-Fusion^®^ HD (Takara Bio Europe, Saint-Germain-en-Laye, France). PTST1 and mCherry were cloned into the vector pUCE-UBI:NOS (Hebelstrup *et al.*, 2010) having a unique *Pac*I restriction site (TTAATTAA) between the promoter (UBI) and the termination site (NOS). Primers to clone the red fluorescent protein (mCherry) were 5'-ATG GTG AGC AAG GGC GAG-3' as forward and 5'-AAT GCT GAG GCA TTA TTA CTT GTA CAG CTC GTC C-3' as reverse. The mCherry sequence was introduced into the plasmid as a marker for visualizing the transgenic tissue in plants. *Ptst1* was amplified from *Hordeum vulgare* leaf-derived cDNA. The primers used were 5'-GGC TGA GGT CTT AAT ATG GAA TGC TTG ACT GCC G-3' as forward and 5'-GCC CTT GCT CAC CAT CTA TTC CAC GGC GAG TTT A-3' as reverse. The primers used for the cloning procedure were designed following the In-Fusion directives.

For the CRISPR/Cas9, a synthetic guide RNA (sgRNA) (5'-CAG AAG TGA ACT TGC TGT TT-3') was designed to target the coiled-coil domain in *ptst1*, while sgRNA (5'-GGC ATG AAC CTC GTG TTC GT-3') was designed to target the first exon in *gbss1a* (Fig. 5). Two sgRNAs were separately cloned into the entry vector pJG85 under the expression of the wheat U6 polymerase III promoter (TaU6). In a second plasmid (pJG80), a wheat codon-optimized Cas9 (TaCas9) was cloned under the expression of the ZmUbi1 promoter. Using Gateway cloning, pJG85 and pJG80 were cloned into the destination vector pANIC6A (Mann *et al.*, 2012).

The plant transformation vectors were subsequently transformed into the *Agrobacterium* strain AGL0 using the freeze/thaw method. Positive colonies were selected on medium with rifampicin (25 µg ml^–1^) and the selection antibiotic of the vectors, namely spectinomycin (50 µg ml^–1^) for the pUCE-UBI:PTST1-mCherry:NOS construct and kanamycin (50 µg ml^–1^) for the CRISPR/Cas9 construct. Independent transgenic barley lines with expression of PTST1–mCherry and PTST1/GBSS1a knock-out lines were generated by *Agrobacterium tumefaciens* (AGL0) transformation following the methods of Holme *et al.* (2012).

### Molecular analysis

Plant materials for molecular analysis were collected from barley (*H. vulgare* cultivar Golden Promise) endosperms of T_0_ and leaves of T_0_ and T_1_ progeny. Genomic DNA was isolated using the CTAB (cetyltrimethylammonium bromide) method (Doyle and Doyle, 1990). Primers were designed to amplify a 560 bp fragment containing the sgRNA. The PCR products were cloned with pCR2.1^®^-TOPO TA vector (ThermoFisher Scientific), and 10 positive colonies were chosen and sequenced. The primers used for *ptst1* amplification were 5'-TGA ATC GCG ATG TAG GTC AG-3' as forward and 5'-GCG CAC TTT GCT ATT GAA TG-3' as reverse. The primers used for *gbss1a* amplification were 5'-CTT CTG GCC TGC TAC CTC AA-3' as forward and 5'-TAG TTG ACG GCG AGG AAC TT-3' as reverse.

Specific primers 5'-CAG TCG CAA ATA AAG GAT GAT GC-3' as forward and 5'-CGA ACA AGA AAA TGC TAA CCT GC-3' as reverse were used to flank 15 bp deletions of PTST1 in barley T_1_ plants. Wild-type plants amplified 221 bp while homozygous mutant plants amplified 206 bp and heterozygous mutant plants amplified both fragments (221 bp and 206 bp).

### Confocal laser scanning microscopy

PTST localization in leaves and endosperms at different development stages of mCherry-tagged PTST and non-transformed plants (cultivar Golden Promise) were observed using an Olympus Fluoview FV1000 confocal microscope as described previously (Hebelstrup *et al.*, 2010). The excitation wavelength for both mCherry and chloroplasts was 559 nm. The fluorescence of mCherry was detected at 580–613 nm and the fluorescence of chlorophyll was detected at 641–681 nm.

### Quantitative real-time PCR

Quantitative real-time PCR (RT-qPCR) was performed as described (Carciofi *et al.*, 2011). Relative quantification of expression was calculated using glyceraldehyde-3-phosphate dehydrogenase (*GAPDH*) as an internal control as described (Pfaffl, 2001). The primers used for PTST1 were 5'-AGA AAT CGC TGT CCA TTG GG-3' as forward and 5'-GCT CCC AGG TCT AAG CTT CA-3' as reverse. The primers used for GBSS1a were 5'-TCA TCT CCG AGA TCA AGG TC-3' as forward and 5'-GAG GTT GAG GAT CCT GGG-3' as reverse. All the analyses were conducted in three technical replicates.

### Starch extraction and amylose content

From three different plants of each independent line that was analyzed we ground 20 T_1_ grains with overexpression of PTST1. We then extracted starch from the flour. Starch extraction and quantification of amylose content were carried out according to the methods described in Carciofi *et al.* (2011).

### Staining of starch in leaves

Leaves from the non-transformed plants (cultivar Golden Promise) and heterozygous *ptst1* knock-out plants were collected after a 12 h light period or a 24 h dark period. Pigments were removed by extraction in 80% (v/v) ethanol at 80 °C for 30 min and then stained by Lugol’s iodine stain solution (stock: 250 mg of I_2_, 2.5 g of KI, 125 ml of ddH_2_O, freshly diluted 1000-fold in 100 mM HCl) for 10 min. The Lugol solution was removed after ~5 min and the leaves were washed briefly with 100 mM HCl to remove the excess iodine. White transmission light was used to observe starch granules using an Olympus Fluoview FV1000 confocal microscope.

### Transient expression and FRET

Transformation of barley endosperm cells was done by biolistics as described before for embryos (Brinch-Pedersen *et al.*, 1996). FRET analyses were carried with an Olympus Fluoview FV1000 confocal microscope using the software supplied by the manufacturer. With this method, FRET is detected by an increase in donor fluorophore [enhanced green fluorescent protein (eGFP)] emission after bleaching of an acceptor (mCherry) fluorophore. For details, see Albertazzi *et al.* (2009).

### Sugar analysis

Grains were collected 15 d after anthesis from both mutants and control plants. The developing endosperms were isolated and powdered after being frozen in liquid N_2_. Sugars were extracted from all samples by incubation with 80% ethanol for 30 min and centrifuged at 80 °C. After centrifugation at 20000 *g* for 5 min, the supernatant was collected and extraction was repeated twice with 10 min incubation time. The supernatants were pooled and dried *in vacuo*. The dried sugars were resuspended in 1 ml of MilliQ water and incubated at 80 °C for 10 min to dissolve dried sugars. Following centrifugation at 20000 *g* for 5 min, the obtained supernatant was analyzed by high performance anion exchange chromatography with pulsed amperometric detection (HPAEC-PAD; Dionex, Sunnyvale, CA, USA). Sugars were identified based on authentic standards sucrose, glucose, fructose, cellobiose, maltose, panose, maltotriose (M3), and maltotetraose (M4). Samples of 5 µl were injected on a CarboPac PA20 column using a flow rate of 0.25 ml min^–1^, 100 mM isocratic NaOH, and the following NaOAc gradient profile: 0–25 min, 13–80 mM linear gradient; 25–30 min, 80–800 mM convex gradient; 30–31 min, 800 mM; 31–32 min, 800–13 mM linear gradient; and 32–40 min, 13 mM. Prior to each injection, a 5 min equilibration with 100 mM NaOH, 13 mM NaOAc was performed. The column was equilibrated by a blank run with the same program as above before the sequence start. Concentration of sugars is based on the integrated peak area of the respective sugar compared with the concentration of the corresponding authentic sugar standard.

### Statistical analysis

All data were subjected to ANOVA using SPSS (Statistical Product and Service Solutions) Version 10.0. The ANOVA mean comparisons were performed in terms of the least significant difference (LSD), at the level of *P*<0.05.

## Results

### AtPTST1 homologs in barley

**Fig. 1.**
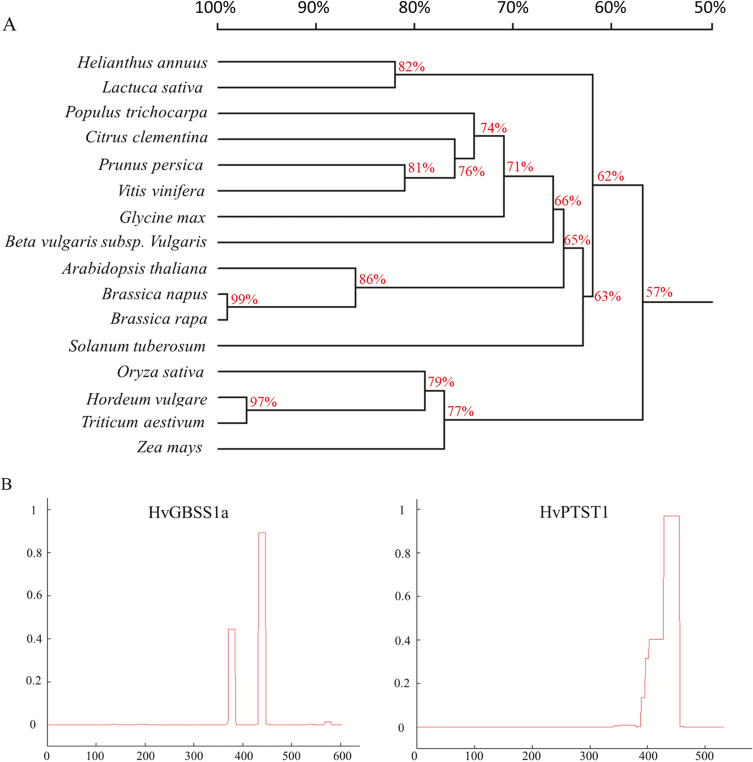
Phylogenetic tree of PTST1 proteins (A) and prediction of the coiled-coil domain (B). The alignment used to generate this tree is available in Supplementary Fig. S1. The coiled-coil domain was predicted by the online tool (https://embnet.vital-it.ch/software/COILS_form.html).

In search of a PTST1 protein in barley, we used BLASTp and, as entry, the amino acid sequence of AtPTST1. Though similar proteins were found in rice, maize, soybean, and other species, no similar protein was found in barley in the NCBI database. However, in the EnSemble Plants database (https://plants.ensembl.org/index.html), a homologous candidate protein was identified (F2EBQ8). Amino acid sequences of homologs of PTST1 proteins from different species were identified in NCBI RefSeq and EnSemble Plants genomes using BLASTp. The candidate PTST1 in barley showed 94% similarity with wheat, 76% with maize and rice, and 54% with Arabidopsis PTST1, respectively (Fig. 1A). We further analyzed the domain structure of the candidate barley PTST1 using UniProt and the COILS/PCOILS www-service and found coiled-coil domains (Fig. 1B, right). They were similar in length to those found in AtPTST1 (Seung *et al.*, 2015). The candidate barley PTST1 also contained an AMPK1_CBM domain functioning as a carbohydrate-binding pocket at the C-terminal end, directly adjacent to the predicted coiled-coils, and it contained an N-terminal transit peptide, which predicts a plastid localization. Therefore, we named this protein HvPTST1. We also identified two coiled-coil domains in HvGBSS1a (Fig. 1B, left). Throughout the rest of this paper GBSS1a and PTST1 refer to the barley homologs unless otherwise indicated.

### Identification of direct interaction between GBSS1a and PTST1 by FRET

**Fig. 2.**
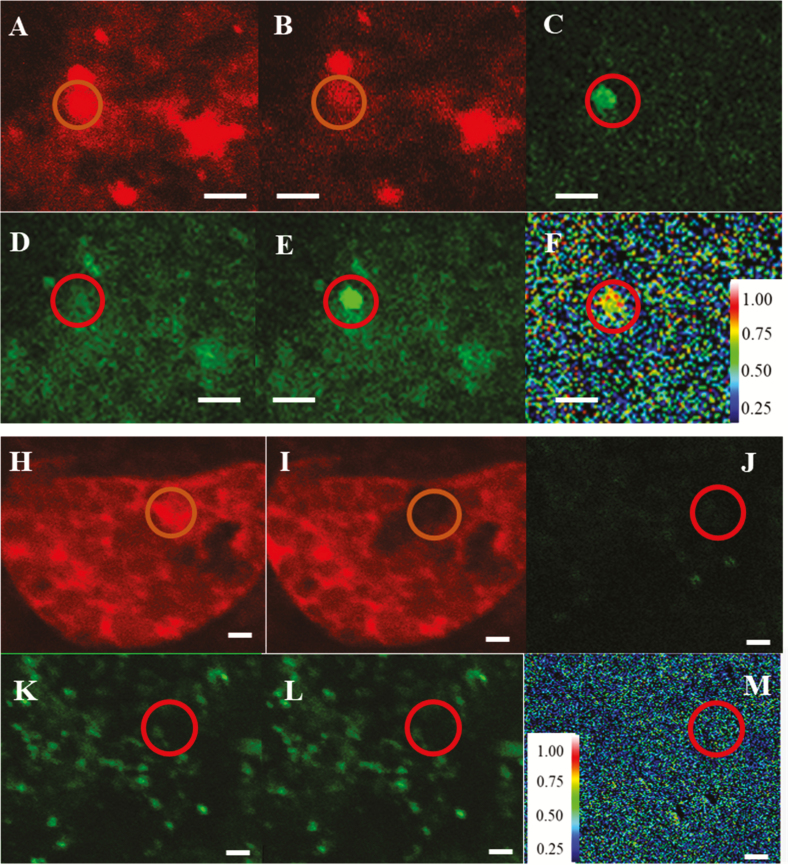
GBSS1a interacts with PTST1 in barley endosperm as confirmed by FRET. Fluorescence images of endosperm expressing: PTST1–mCherry and GBSS1a–eGFP (A–F), and mCherry and GBSS1a–eGFP for negative control (H–M). Excitation and emission wavelengths of eGFP were 488 nm and 500–545 nm, respectively (D, E, K, L); excitation and emission wavelengths of mCherry were 559 nm and 570–670 nm, respectively (A, B, H, I). Confocal laser scanning microscopy of endosperm before (A, D, H, K) and after (B, E, I, L) bleaching. (C) Difference of green fluorescence between (D) and (E). (J) Difference of green fluorescence between (K) and (L). (F, M) FRET efficiency between GFP and mCherry. Scale bar=3 µm.

To test the interaction between GBSS1a and PTST1, we transiently expressed the proteins in barley endosperm by biolistic co-bombardment with GBSS1a–eGFP and PTST1–mCherry vector DNA. Direct interaction was scored by FRET. Co-bombardment of the outer endosperm with GBSS1a–eGFP and mCherry vector controls was used as control for non-interaction. PTST1–mCherry was observed in subcellular compartments (Fig. 2A), which is in agreement with a plastidial localization. This corresponds well with its N-terminus having a predicted transit peptide domain. In contrast, the vector control mCherry was localized in a pattern in the endosperm cells in agreement with a cytosolic localization (Fig. 2H). Comparing Fig. 2A (before bleaching) and Fig. 2B (after bleaching), there was a region (orange circle) of the endosperm where the acceptor (PTST1–mCherry) was bleached while no changes occurred outside of the bleached area. Donor fluorescence (GBSS1a–eGFP) increased from before bleaching (Fig. 2D) to after bleaching (Fig. 2E). This difference is shown in Fig. 2C. FRET efficiency is shown in Fig. 2F, confirming direct interaction between the donor and acceptor. For the control experiment, Hordein–mCherry red emission disappeared by bleaching (Fig. 2H, I), but did not result in stimulation of GBSS1a–eGFP (Fig. 2J, K, L), confirming that no FRET could be detected (Fig. 2M).

To detect if PTST1 and GBSS1a are expressed in the same tissue, gene expression of PTST1 and GBSS1 was measured in endosperm, leaf, root, and anther. Both genes had a high expression level in endosperm and anthers (Supplementary Fig. S2).

### PTST localization in chloroplasts and endosperms

**Fig. 3.**
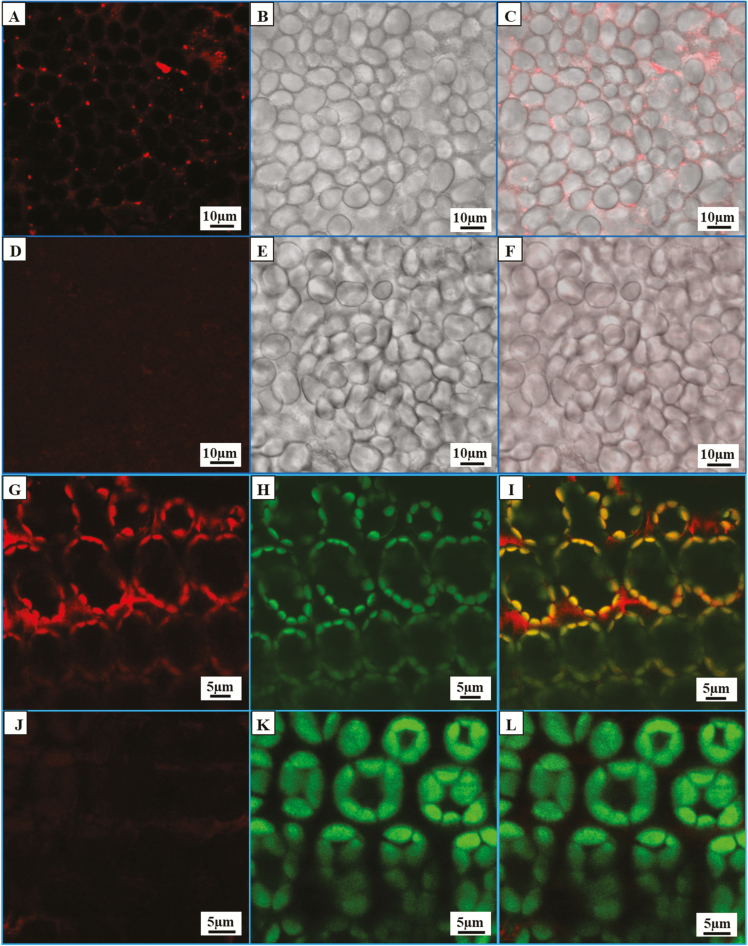
Localization of fluorescently tagged PTST (PTST–mCherry) in endosperm and leaves. (A, D, G, J) mCherry channel. (B, E) Transmission channel. (H, K) Chloroplasts were detected by their red autofluorescence and were dyed by green to distinguish them from mCherry. (C, F, I, L) Chanel overlays. (A–C, G–I) PTST–mCherry overexpression plants. (D–F, J–L) Wild-type control plants. PTST–mCherry was located as scattered dots in endosperm (A–C) and in chloroplasts in leaves (E). No mCherry fluorescence was observed in wild-type plants (D, J).

To visualize the localization of PTST1, permanent transgenic plants with ectopic expression of PTST1–mCherry were made by transformation of developing embryos and regeneration of plants from the embryonic callus. Using confocal laser scanning microscopy, we localized PTST1–mCherry inside endosperm cells (Fig. 3A, C; Supplementary Fig. S4) and in leaf chloroplasts (Fig. 3G, I), while no mCherry fluorescence was observed in non-transgenic plants (Fig. 3D, J). Chloroplasts were identified by autofluorescence from chlorophyll (Fig. 3H, K). In leaf, PTST1–mCherry showed a distribution throughout the chloroplast in agreement with a stromal localization (Fig. 3G, I). In endosperms [10–25 days after anthesis (DAA)], PTST1–mCherry was observed as scattered dots in between the starch granules rather than bound to starch (Supplementary Fig. S4). The morphology of starch granules in the endosperm of plants with PTST1 overexpression was similar to that of non-transgenic plants.

### Amylose content in PTST1 overexpression plants

**Fig. 4.**
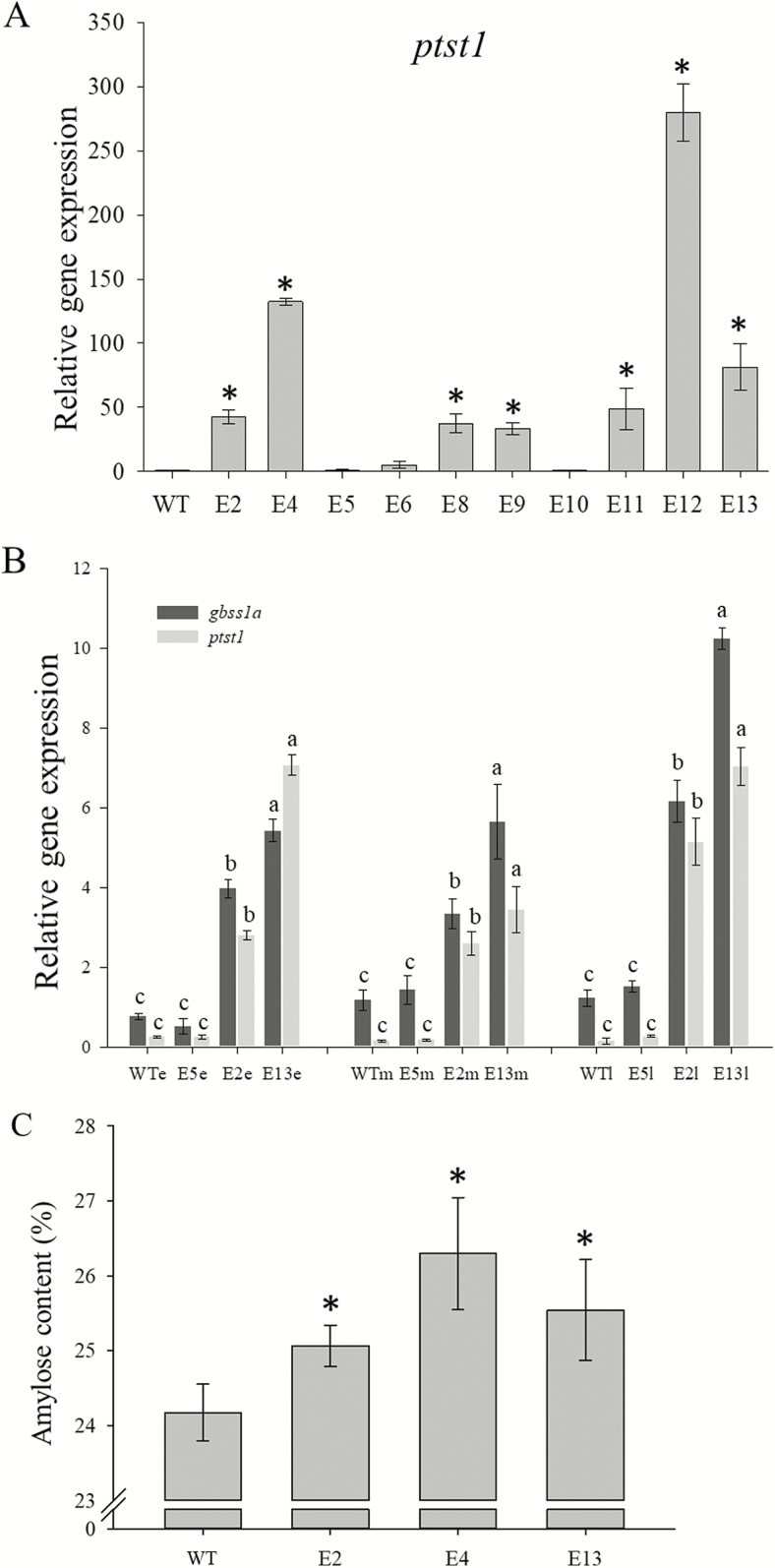
Relative gene expression of *ptst1* in leafs from T_0_ wild-type Gold Promise (WT) and *ptst1* overexpression plants (A), *ptst1* and *gbss1a* gene expression in T_1_ endosperm (B), and their amylose contents (C). e, m, and l represent early, middle, and late filling stage, respectively. Error bars represent the SDs of biological replicates (*n*=3). Significant differences (*P*≤0.05) are indicated with letters in (B) and with * in (A) and (B) to indicate significant differences from the wild type.

We produced 10 independent lines of T_0_ plants, and 7 of them showed overexpression of *ptst1* in leaves. The level of *ptst1* mRNA was 33 (E9) to 279 times (E12) higher than in non-transgenic plants (Fig. 4A). We chose two lines with different levels of *ptst1* up-regulation (E2 and E13), Golden Promise (non-transgenic plants), and E5 (transgenic false positive) to check the expression of *gbss1a* and *ptst1* in the T_1_ endosperms of early, middle, and late developing stages. The expression of *gbss1a* was significantly higher in lines with *ptst1* overexpression (E2 and E13; Fig. 4B) as compared with non-transgenic control. We also observed a higher protein level of GBSS in purified starch granules from *ptst1* overexpression plants compared with those of wild-type plants (Supplementary Fig. S5; Supplementary Table S1).

To test if up-regulation of *gbss1a* expression increases amylose content, we selected three independent lines of *ptst1* overexpression plants to evaluate the amylose content in mature grain. All three lines showed a small but statistically significant increase in amylose content compared with that of the wild type (E4) (Fig. 4C).

### *ptst1* and *gbss1a* mutants generated by CRISPR/Cas9

**Fig. 5.**
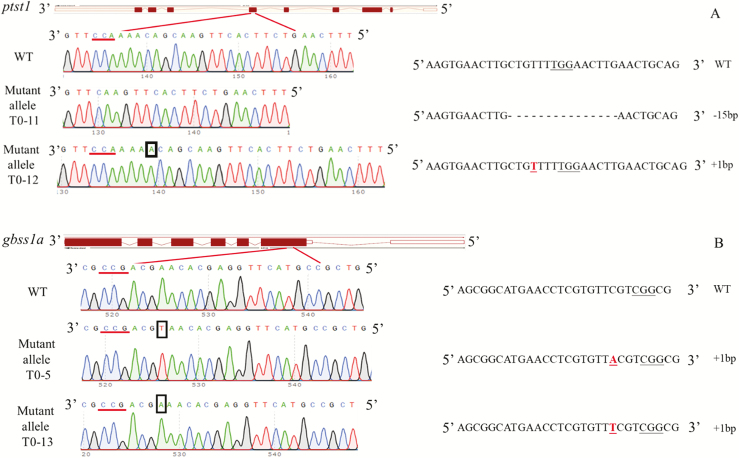
Mutations of the *ptst1* or *gbss1a* genes induced by CRISPR/Cas9. The wild type (WT) sequence around the CRISPR/Cas9 target of the genes PTST1 and GBSS1a were identified by direct sequencing. These are shown in each panel: (A) PTST1 and (B) GBSS1a. For each of the genes, we identified at least two mutant alleles from independent lines by direct sequencing: of PTST1 (mutant alleles T0-11 and T0-12) and of GBSS1a (T0-5 and T0-13). Nucleotide insertions in mutant alleles are indicated by underlining (right) or with boxes (left), and the 15 bp deletion in the mutant allele T0-11 is indicated as a gap. The CRISPR/Cas9 PAM motifs are underlined. Note that the DNAs were sequenced in the reverse strand (shown to the left) of the reading frame of the genes (as shown to the right).

Primary transformants (T_0_) were generated by *Agrobacterium*-mediated transformation with the CRISPR/Cas9 constructs as illustrated in Fig. 5. A total of nine and eight hygromycin-resistant T_0_ plants were generated from 217 or 460 embryos transformed with the *gbss1a* knock-out or *ptst1* knock-out CRISPR/Cas9 constructs, respectively (Supplementary Table S2). Mutations were identified by PCR and sequencing. Heterozygous plants with inserts or deletions were identified by their double peaks in the sequencing chromatograms (Supplementary Fig. S6) while the wild types showed clear single peaks (Fig. 5). PCR products from mutated plants were further TOPO cloned to identify individual alleles. From each T_0_ plant, 10 TOPO clones were picked and sequenced. If all 10 clones showed the same insertion/deletion, the plants were identified as candidate homozygous mutants. If the plants showed a combination of the wild type and insertions/deletions, the plants were identified as heterozygous mutants. Plants showing more than two different alleles were considered mosaic (Supplementary Table S2). Based on these sequences, five T_0_ plants with *gbss1a* mutations and six T_0_ plants with *ptst1* mutations were heterozygous (Table 1). All the *gbss1a* mutations had 1 bp insertions. Four heterozygous *ptst1* plants had the same 15 bp deletion and the other two had 1 bp insertions (Fig. 5). Progeny of T0-10 and T0-11 *ptst1* plants, that were all of the 15 bp deletion type, were subsequently genotyped by PCR and agarose gel electrophoresis based on the 15 bp difference from a wild-type allele (Supplementary Fig. S3) to analyze the segregation ratio.

**Table 1.** Segregation ratio of the starchless phenotype and the genotype in the T_1_ seedling generation that were progeny of T_0_ heterozygous *ptst1* mutant plants

	Phenotype			Genotype			
	WT	Starchless	Segregation ratio	WT (+/+)	+/–	–/–	Segregation ratio
T1-10	144	51	2.82:1	17	36	0	1:2.1:0
T1-11	157	54	2.91:1	42	80	0	1:1.9:0
T1-12	173	55	3.15:1	ND	ND	ND	–

Genotypes of T1-10 and T1-11 were identified by PCR basing on the 15 bp deletion. The genotypes of T1-12 were not determined because there is only a 1 bp insertion without a restriction site.

### Grain phenotypes of *ptst1* and *gbss1a* loss-of-function mutants

**Fig. 6.**
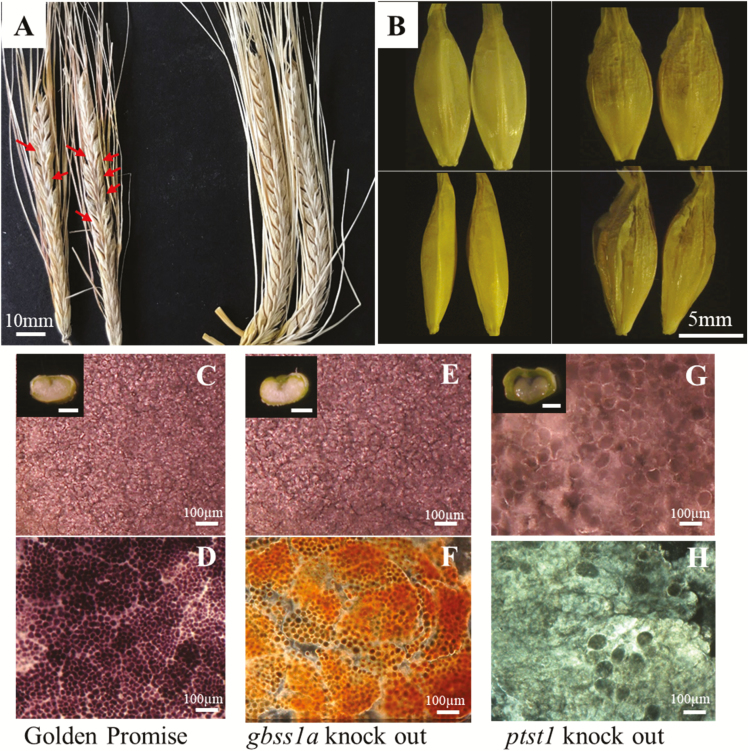
Phenotype of *ptst1* knock-out endosperm. (A) Spikes of heterozygous *wt/ptst1* (left) mutants and homozygous *gbss1a* (right) mutants. Red arrows indicate starchless grains. (B) Phenotype of mature starchless grains of *ptst1* (left) and grains of *gbss1a* (right) mutants. The images were recorded to show the top view of the grains (upper panels) and the side view (lower panels). (C, E, G) Cells of the inner endosperm of developing grain shown with dark field light microscopy. Images at top left corner of each figures are transections of developing endosperms. Scale bar=1 mm. (D, F, H) Starch granules after iodine staining of the inner endosperm shown with dark field light microscopy. Starch granules are present in Golden Promise control (D) and in *gbss1a* knock-out (*waxy*) but absent in *ptst1* knock-out endosperm cells.

We analyzed the endosperms of heterozygous *ptst1* and *gbss1a* T_0_ plants at 15 DAA. All the endosperms of *gbss1a* homozygous knock-out plants developed starchy endosperm. Of the two *ptst1* heterozygous knock-out lines, ~25% of the grains developed starchless wet endosperm (Fig. 6G, insert left corner panel). To analyze if there was any starch in these developing grains, we used iodine staining, which gives a distinct staining of starch granules. Unstained endosperms of developing wild-type and *gbss1a* mutant grains contained identifiable starch granules (Fig. 6C, E; Supplementary Fig. S8) while the *ptst1* mutant grain endosperm only had starchless cells (Fig. 6G). With staining, the starch granules of the wild type showed a distinctive dark purple color characteristic for wild-type starch (Fig. 6D). The starch granules of *gbss1a* knock-out endosperms had a yellow-brown color (Fig. 6F), in agreement with the waxy phenotype expected by GBSS1a loss of function. Staining of *ptst1* knock-out endosperm further confirmed that they were starchless (Fig. 6H). After maturation, the starchless grains from the heterozygous *ptst1* knock-out lines contained only bran (Fig. 6A, indicated by red arrows; Fig. 6B, left panels). *Gbss1a* knock-out grains were all fully developed (Fig. 6A, right; Fig. 6B, right panels), The T_1_ grains from three independent heterozygous T_0_ plants (two with the 15 bp deletion, and one with a 1 bp deletion) were collected and the ratios of starchless/normal starch grain phenotype was scored (Table 1). The starchless trait was segregating in a classic 1:3 Mendelian ratio. To analyze the segregation of the genotype, we isolated genomic DNA from developing endosperms of T_1_ grains. We were unable to obtain genomic DNA from starchless grains. Also, the *ptst1* knock-out grains with starchless endosperms did not germinate. Therefore, we could only genotype endosperms that showed a wild-type phenotype, and they were either heterozygous or wild type with a segregation ratio of 1:1.9 (Table 1; Supplementary Fig. S3). No homozygous mutant genotype was found among those.

### Sugar analysis of *ptst1* loss-of-function mutant sap

We collected developing endosperm from the two independent starchless *ptst1* mutants and from wild-type grains to identify their sugar content. Six types of sugars were identified in both mutants of starchless *ptst1* grains, while eight types of sugar were found in wild-type grains (Supplementary Fig. S7). In two *ptst1* mutant grains, the most abundant types of sugar were fructose and glucose, accounting for 68% and 83% of the total identified sugars, respectively (Table 2). Some cellobiose and maltose and a small amount of sucrose and panose were identified, while no M3 and M4 were found in *ptst1* mutant endosperm. Following treatment with amyloglucosidase, we identified unknown components as peaks that did not disappear as an effect of the treatment. This accounted for ~1/6 of the *ptst1* mutant grain extract. The unidentified peaks that disappeared with amyloglucosidase and were converted to glucose were defined as branched maltooligosaccharides, accounting for ~1/3 of the *ptst1* mutant grain extract. The sucrose content was higher in the wild-type endosperm, while the glucose and fructose contents were significantly lower in wild-type grain in comparison with the *ptst1* mutant grains. Minor amounts of M3 and M4 were observed in the wild type but not in the mutants. Around 1/2 and 1/9 of the extract of wild-type grains were unknown components and maltooligosaccharides, respectively.

**Table 2.** Sugar analysis of *ptst1* mutant endosperm

Type of sugar (µg per grain)	Mutant 1	Mutant 2	Wild type
Sucrose	7.0 ± 1.0	6.0 ± 9.7	142.3 ± 14.7
Glucose	85.3 ± 11.2	113.7 ± 24.5	29.5 ± 2.3
Fructose	91.4 ± 13.7	122.9 ± 19.5	74.2 ± 9.9
Cellobiose	41.6 ± 9.3	26.0 ± 10.4	8.8 ± 1.3
Maltose	24.2 ± 9.7	12.8 ± 2.4	37.5 ± 3.6
Panose	10.2 ± 4.0	8.2 ± 0.4	9.9 ± 1.2
Maltotriose	0	0	6.9 ± 1.4
Maltotetraose	0	0	74.5 ± 14.5

Data are shown as mean ±SD. Mutant 1 and mutant 2 are grains from two different *ptst1* knock-out lines. Wild type is the grain at the same development time of Golden Promise.

### Starch levels in heterozygous *ptst1* leaves

**Fig. 7.**
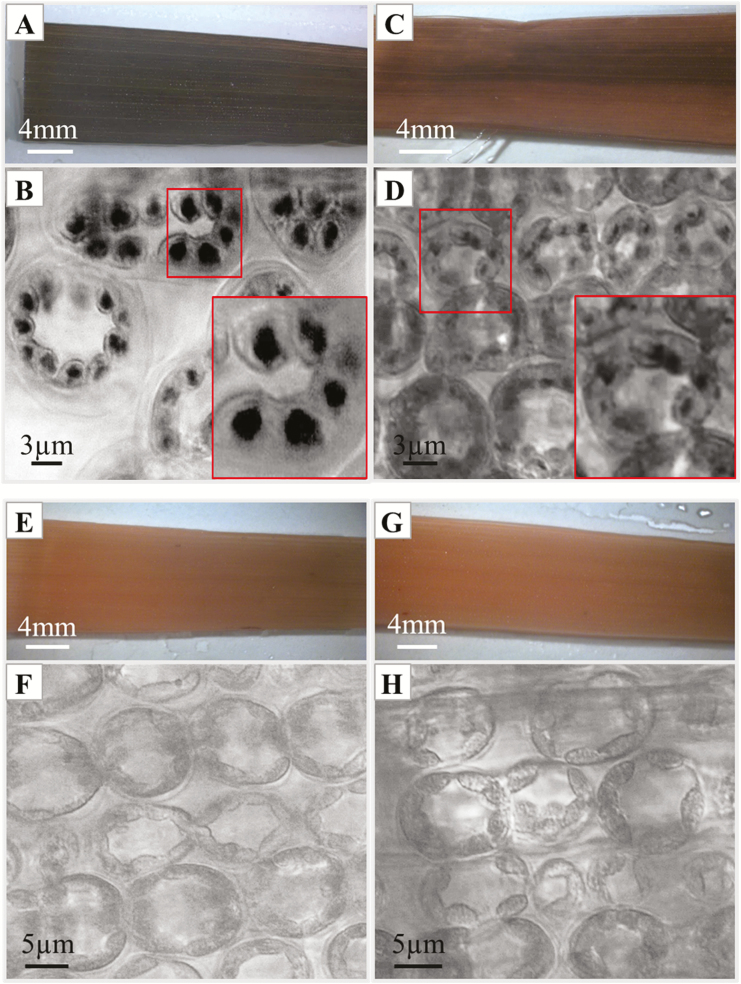
Qualitative identification of relative starch levels in leaves after an extended 24 h dark period or at end of day (12 h light) by iodine staining. (A, B) 12 h light control Golden Promise leaves. (C, D) 12 h light heterozygous *ptst1* knock-out leaves. (E, F) 24 h dark control Golden Promise leaves. (G, H) 24 h dark heterozygous *ptst1* knock-out leaves. (B, D, F, H) Confocal laser scanning microscopy of iodine-stained starch granules (transmission channel). Red boxes indicate enlargements.

To test if *ptst1* affected the starch accumulation in leaves, we did a qualitative score of relative starch levels in leaves that were kept either for 24 h in dark (extended dark period) or for 12 h in light (end of day) by iodine staining (Fig. 7). At the end of the extended dark period, no starch was observed in wild-type or heterozygous *ptst1* leaves (Fig. 7E–H). However, at the end of day, wild-type leaves accumulated more starch (Fig. 7A, B) than heterozygous *ptst1* leaves (Fig. 7C, D). The central part of each chloroplast in mesophyll cells of the wild type contained one or more larger starch granules, while in the *ptst1* knock-out starch granules were smaller and some chloroplasts were devoid of starch granules.

## Discussion

### PTST1 is localized around starch granules in the barley endosperm

Starch synthases and other enzymes that are involved in starch biosynthesis and degradation are dependent on direct interaction with α-glucan structures. This is efficiently achieved through CBMs, in this case also known as starch-binding domains. In Arabidopsis, starch-branching enzymes, starch debranching enzymes, as well as members of the PTST family all have CBM48 domains. In HvPTST1, we identified an AMPK1_CBM domain, which showed high similarity to CBM20, CBM48, and CBM53, indicating a role in starch binding. By tagging GBSSIa with eGFP, we have previously seen that HvGBSSIa–eGFP is bound to starch granules and concentrated in internal concentric spheres (Hebelstrup *et al.*, 2017). However, PTST1–mCherry was localized throughout the chloroplast in leaves (Fig. 3H–I) and in close contact with starch granules in developing endosperm (Supplementary Fig. S4). The FRET studies indicated a direct interaction of GBSSIa and PTST1 in barley endosperm. Therefore, the localization of PTST1 was in accordance with a function as a starch synthase cofactor cycling between starch granule surfaces and stroma, and which interacts only transiently with starch during the GBSS localization (Seung *et al.*, 2015).

### PTST1 overexpression increases the content of amylose in grain endosperm

The commercial interest in starches with modified amylose content has driven the application of biotechnological tools to alter amylose content *in planta*. Four factors affecting amylose biosynthesis and content have been suggested as targets: the amount of GBSSI protein, the availability of ADP-glucose and maltooligosaccharides, and the physical space available within the matrix of amylopectin (Smith *et al.*, 1997). Overexpression of GBSS has been reported in potato tubers and wheat, where amylose levels were unaffected by the overexpression (Flipse *et al.*, 1996; Sestili *et al.*, 2012). Here, we observed an increased amylose content in mature barley grains with overexpression of PTST1. This finding confirmed the role of PTST1 in amylose biosynthesis, which was also indicated by the interaction between PTST1 and GBSS1a (Fig. 2). Therefore, the interaction between PTST1 and GBSS1a could be an additional factor or target to modify amylose levels.

### PTST1 is essential for starch biosynthesis in barley storage tissue

Even though around half of the T_1_ grains (that were progeny of heterozygous plants with a *wt/ptst1* genotype) had a heterozygous genotype, no homozygous *ptst1* plants was found in either the T_0_ or T_1_ generation (Table 1). Some endosperms of T_1_ grains showed a unique phenotype with only sugar and no starch being accumulated during development (Fig. 6G). These data indicate that *ptst1* homozygous endosperm dries out during the desiccation stage. Unfortunately, we could not extract genomic DNA from developing homozygous mutant grains so the mutant phenotype could not be matched with a homozygous genotype directly. Reverse proof was provided by the observation that genotypes of wild phenotype endosperms were either wild type or heterozygous with a segregation ratio of 1:2 (Table 1), indicating that the starch-free endosperms could be homozygous mutants. In addition, fewer hygromycin-resistant T_0_ plants of *ptst1* knock-outs (2.2%) were obtained than of *gbss1a* knock-outs (6.0%). We obtained two homozygous *gbss1a* knock-out plants, while no homozygous *ptst1* knock-out plants were found in the T_0_ generation. It is possible that homozygous *ptst1* mutant seedlings regenerated from callus do not survive. This is substantiated by the low regeneration frequency (1.7%) of plants with CRISPR/Cas9 targeting of the PTST1 gene. We did not observe a dramatic increment of glucose in *ptst1* mutant endosperms. One explanation is that when on the same spike there are mutant, heterozygous, and wild-type endosperms with a segregation ratio of 1:2:1, the glucose is transported to the other endosperms when the content in mutants is saturated. Our data showed that PTST1 is also expressed in barley leaves, indicating that it may have a function there.

It has been reported that SS4 is critical for starch granule initiation in both storage and transient starches (Toyosawa *et al.*, 2016). Since SS4 itself in Arabidopsis does not possess a specialized CBM, the interaction with PTST2 provide access to a CBM that can bind suitable MOS substrates to initiate starch granules biosynthesis (Seung *et al.*, 2017). In barley, a homologous gene of *ptst2* was found and called *fra* (Saito *et al.*, 2018). However, the *fra* mutant only produced fractured starch granules and an opaque phenotype rather than a decrease in starch granule number, suggesting that FRA does not interact with SS4 to mediate starch granule initiation in barley endosperm. Therefore, the starch-free phenotype we found for the *ptst1* mutant in barley could be due to the interaction between PTST1 and SS4. In Arabidopsis leaves, PTST1 only interacts with GBSS1a and the *ptst1* mutant produces amylose-free leaves (Seung *et al.*, 2015). However, in barley endosperm, we have demonstrated that *ptst1* plays a crucial role for development of starchy endosperm since the *ptst1* mutant produces non-viable grain with starch-free endosperm. Extensive screens for amylose-free mutants have been conducted in barley, but no *ptst1* mutant alleles have so far been isolated. Our data suggest that it is because this mutant is lethal.

## Supplementary data

Supplementary data are available at *JXB* online.

Table S1. GBSS content in starch granules.

Table S2. Transformation efficiency of barley embryos of *gbss1a* and *ptst1* by CRISPR/Cas9.

Fig. S1. Accession numbers and sequences.

Fig. S2. Expression of *gbss1a* and *ptst1* in different tissues of barley.

Fig. S3. Genotyping of *ptst1* T_1_ plants with a 15 bp deletion.

Fig. S4. Localization of PTST1–mCherry in developing endosperm cells.

Fig. S5. SDS–PAGE of purified starch granules.

Fig. S6. Sequencing of heterozygous mutants of *ptst1* (A) and *gbss1a* (B).

Fig. S7. HPAEC-PAD chromatogram for the extract of *ptst1* mutant and wild-type endosperm.

Fig. S8. Confocal micrographs of cross-sections of developing endosperm.

## Supplementary Material

Supplementary data
