# Supplementary material for: Protein Targeting to Starch 1 is essential for starchy endosperm development in barley
Source: J Exp Bot. 2018 Nov 8;70(2):485–96. doi: 10.1093/jxb/ery398 (PMC6322578; doi:10.1093/jxb/ery398)
Supplement: Supplementary data [file ery398_suppl_supplementary_tables_s1-s2_and_figures_s1-s8.pdf]

Table. S1 Relative levels of GBSS content in starch granules determined by intensity of GBSS bands in Fig. S5

	GBSS	
Golden Promise	1.00±0.07[b]	0.28±0.06[a]
PTST-mCherry	1.27±0.05[a]	0.30±0.03[a]

Table S2 Transformation efficiency of barley embryos of *gbss1a* and *ptst1* by CRISPR-Cas9

	No. of transformed embryos	hygromycin resistent T0-plants	homozygous	heterozygous	mosaic
gbss1a	217	9 (4.1%)	2 (0.9%)	5 (2.3%)	2 (0.9%)
ptst1	460	8 (1.7%)	0	6 (1.3%)	2 (0.4%)

Fig. S1 Accession numbers and sequences

Fig. S2 Expression of *gbss1a* and *ptst1* in different tissues of barley. Endosperm10, 20, 30 represent the developing endosperms at 10, 20 and 30 days after anthesis, respectively. AntherA and antherB represent young and old anthers, respectively.

Fig. S3 Genotyping of *ptst1* T_1-plants with 15-bp deletion. NC and PC represent negative control and positive control, respectively. Negative control is the PCR product from golden promise and positive control is the plasmid of clone with 15-bp deletion.

Fig. S4 Localization of PTST1-mCherry in developing endosperm cells. PTST1-mCherry (A) and transmission light (B) showing starch granules in amyloplasts were recorded as described in Fig. 3. Overlays are shown in (C).

Fig. S5 SDS-PAGE of purified starch granules. Protein from 5 mg of purified starch granules from golden promise (lanes 1-3) and PTST1 overexpression grains (lane 5 – 7) were analyzed by SDS-PAGE. GBSS was identified at the expected size. Starch from PTST1 overexpression grains had a higher content of GBSS than that of golden promise grains.

Fig. S6 Sequencing of heterozygous mutants of *ptst1* (A) and *gbss1a* (B). The insertion of a T in the *ptst1* mutant allele T0-12 and A in the *gbss1a* mutant allele T0-5 could be identified in PCR products from heterozygous plants by introduction of double peaks in the chromatogram down stream of the inserted nucleotides (marked with boxes).

Fig. S7 HPAEC-PAD chromatogram for the extract of mutant and wild type endosperm. The standards used for identifying sugars comprises (1) sucrose, (2) glucose, (3) fructose, (4) cellobiose, (5) maltose, (6) panose, (7) M3 (trisaccharide) and (8) M4 (tetrasaccharide) as indicated in (A).

Fig. S8 Confocal micrographs of cross sections of developing endosperm. Golden promise (A-D) and *ptst1* mutant (E-H). Three different channels were recorded: blue autofluorescence, mostly from cell walls (A, F), red autofluorescence from chlorophyll (B, F), and transmission light (C, G). Chanel overlays are shown in (D, H). Endosperm is marked with 'e' and pericarp with 'p'.

Fig. S1 Accession numbers and sequences

> Helianthus annuus XP 021983041
MECHTMSTSIQGLSSCQRGNRHISWCYHYNSAPLSLRKNFWKLSHCMKNGRHYAHSRV
LCLPTTLEEEFSSMQSKNYSKDDKYASDSFSEDVSEQLVDSDQLKSLIADAERAKLLKKL
SEANQHNRYLKRQLIVREEAFAQFKSELAVTELEIQALLSMAKEIASYGIPAGSRKINGK
YIQSLLLLQLQGMQEKLKKQIKDVELAQSKEVTLSWSGMAESVQVMGSFDGWSHGEY
LSAEYTGSYTRFSTSIMLRPGRYEIKFLVDGEWLLSPEYPTVGEGLMENNLLVVE

> Populus trichocarpa XP 006383327
MEIGTTRCCIGKQVPWFSRNSRMLDVESIHKLPYHVFGCTIKPSNLRFAHSYWGSARKN
WMSNVLWRTYTMPVSLEESSSEQPEDYSDDEGSSSEDLPEEPLDQSLSSDELKALLLDS
QRKNLTKKLSEANQQNRFLKRQLHVMEDALVNFKSELAVMELEIQALVTLSEEIAQYEI
PEGSRKINGKYVQSHLLSRLRVLQEKLKEQIKDVDAAKSKEVSLFWCGMAESVQVMGS
FDGWSQGEHLSPEYDGSFTKFSATLMLRPGRYEIKFLVDGDWQLSPEYPTIGEGLTENN
LLIVE

> Citrus clementina XP 006482134
MEIGTARGCLDKTVSYFSRNLSELGWKNARKVSYPVAAAWNLRTSYQKLAHEHIVSAR
IRRHHRSSRLLWRAYSMPVSVAADRAEDAEDDSEDSPQRILSQPLSNDELMSLLADSER
KKLMTKLSEANQQNRFLKRQLHIKEDALVDFKSELAVMELEMQALVSLAEEIAQSGIPE
GSRKINGKFIQSHLLSRLEAVHEKLKEQIKDVIAVQSKEVPLFWCGMAQSVQVMGTFDG
WSQGEHLSPEYNGSFTKFSTTLMLRPGRYEIKFLIDGEWQLSPKFPQFGEGIMENYLLIVE

> Lactuca sativa XP 023729179
MECYTMSTSIQGLLSGRCRNKQISWILWPNRSIHGFQYIRNVHRYPHCRLLCFPPTLNEQ
SSSLQSKKNSNGDDEHVLEQPIDNDQLKALLADAERAKLLRKLSEANQHNRYLKRQLL
VKEDALAEFKSELAVTELEIQGLLNMAKEIVSYGIPAGSRKINGKYIQSLLLLQLQGVQE
KLKKQIKEVELAQSKEVSLHWYGMAESVQVMGSFDGWSHGEDLSAEYTGSYTSFSTSI
MLRPGRYEIKFLVDGEWALSPEYPTVGEGLMENNLLIVE

> Oryza sativa XP 015627711

MECLTTSFTRNPGREYNLICPSEALSEKQRIQRRVLCYFPASTNSRRCRKFTTMAYPVSPI
AGRRSNWRSFAASLNLEDGPASSDSTSSPSEQTSDGGEVYGDPSENLNSRKLKSDELKSL
LADSERSKLLKKLSEANQYNRFLKRQLQMKDNDVVKFKSELAVMELELQALVALAEEI
ANFDVPSGSRKINGKYIQSHLLTRLEAVHDKVMEQIKDVDSLKHQEISVFWVGIAENVQI
MGSFDGWSQGEAMSMEYSGYQARFSATLNLRPGRYEIKFLVDGEWRLSLEYPIDGEGS
MQNNILVVN

> Hordeum vulgare F2EBQ8 (UniProtKB)
MECLTAGFAARSVGREYNFVCPSKPVGEKQWVPGRVLCYFTAHTNSSRCCKVATGVCP
ISPVVGRRSRWRSFAASLNLENGPAPSSSTLSSSGQTSEQPTSAELKSLLADKERSKLLRK
LSEANQHNRFLKRQSQIKDDAAVKFRSELAVLELELQALVGLAEEIANFDIPAGSRKVN
GKYIQSHLLSRLEAVHDKVMVQIKDIESLRPREIAVHWVGMAENVQIMGSFDGWSHGE
AMSREYSGDYARFSATLKLRPGSYEIKFLVDGEWKLSSEYPITGEGLTQNNKLAVE

> Arabidopsis thaliana NP 568573
MGCVPRIEFGCSSQSLTLSWNLRAWNLCRLNTISHFQKLPYPLVASTRKHYKNSLLLKR
FLVGVGTEESSLSEDLLDESLSRPLTSDELKSLLIDTERSKLVKKLSEANQQNRFLKRQLK
TQEHEITNIKTELALMELEVQALVKLAEEIANLGIPQGSRKISGKYIQSHLLSRLDAVQKK
MKEQIKGVEAAQSKEVHVFWIGMAESVQVMGSFDGWSQREDLSPEYSALFTKFSTTLF
LRPGRYEMKFLVDGEWQISPEFPTSGEGLMENNVLVVE

> Beta vulgaris subsp. Vulgaris XP 010670151
MACHAIGLVRYFPDEPVLCLSWSSIKSNMNVYGVRCNLASKALGAGFHNLSGLQRISTG
RYTPFHSSWRTYSTSLGLEEQSSSGSSEFQGDETDFADPAEEQRPRPLSRNELKTLLADSE
RSKLVKKLSEANQHNRFLKRQLQGREEALVNFKGDLAVLEREIQALVALADEITKAGIP
EGSRKINGKYIHSHLISRLKTVQHKLQNQIKDVVVAQSKEVQVYWIGMAESVQVMGSF
DGWSQGEHLSPEYTGSYTKFSTTLLLRPGRYEIKFLVDGEWQISPEFPTIGEGITENNLLV
VE

> Brassica napus CDY30270
MKHFCTSQGCVPRMEFGFLSNLFDTDLVHVPVLVLVLEHRHCLEQNLTLSWNLKASSL
GSLATISHLQRLPLSMVASSRNHYKNSLLLKRYLVRVGSTEEEHSLSEDSLDDSVSRPLT
SDELKSLLIDNQRSKLVKKLSEANQHNRFLKRQLKTQEDEITSIKSELAIMELEVQALVN

LAEEIANLGIPQGSRKISGKYIQSHLLTRLDAVHNKLKEQIKDVEAAQSKEVNVFWIGMA
ESVQVMGSFDGWSQPEDLSPEYTASFTKFSTTLVLRPGRYEMKFLVDGEWQISREFPTS
GEGMLENNVLVVE

> Brassica rapa XP 009139925
MEFGFLRHCLEQNLTLSWNLKASSLATTSYLQRLPLSMVASSRNHYYKNSLLLKRYLV
RVGSSTEEEHSLSQDSLDDSVSRPLTSDELKSLLIDNQRSKLVKKLSEANQHNRFLKRQL
KTQEDEITNIKSELAIMELEVQALVNLAEEIANLGIPQGSRKISGKYIQSHLLSRLDAVHN
KLKEQIKDVEAAQSKEVNVFWIGMAESVQVMGSFDGWSQPEDLSPEYTASFTKFSTTL
VLRPGRYEMKFLVDGEWQISREFPTSGEGMLENNVLVVE

> Glycine max XP 014626709
MEISIARSHLETQGIFFSNISRTIGWESIRKLPCNVAAQGSRRDFHRLASSCQAFTAVYPRR
SFICRANSMPISLQESASYGDNSIEDEDPYTDLEEEALAKPPTSEQIMTLLADTQRAKLTK
KLSEANQQNRFLKRQLNVKEDALVKFKSELAVMELEIQALVRLAEEIAQCGIPEGSRKIN
GKYIHSHLVARLEAVNELLKEQIKDVDAAQSKEVSVFWVGMAESVQVMGTFDGWSQG
EHLSPEYTGSYTRFSTTLLLRPGRYEIKFLVDGEWKLSPEFPIIGEGLTKNNLLVVE

> Prunus persica XP 020424944
CSLDNQVLWLSRESSRKLDWRNIHPLPHIVTTWKLKVGYQRLIDCQVSTGKHSLNHVF
WRRHSIHTSLEESSSVQGETYSGNDEYAHKDSQENHLSQPLRSNELKLLLADSERTKLIK
KLSEANQQNRFLKRQLHIKEDALVNFKSELAVLELEIQALVKLAEENTKSVIPQGSRKIN
GKYIQSHLLSRLEAVHEKLKEQIKDVDAVQSKEVPLFWYGMAESVQVMGTFDGWSQG
EHLSSEYTGSFTAFSTTLMLRPGRYEIKFLVDGEWKLSPEFPTVGEGLMKNNLLIVE

> Solanum tuberosum XP 006357768
MASYNSRKGFHRMHKSFSNPIPCTSWKIFCAPENLEKRFSVVVSEKIADAGLSDPEQPLR
SEELRLLLADAERSKLLKKLSEANRYNRLLKRELQAKEDALVNFKSELSVTELEIQVLAR
LAEEIAKSAIPAGSRKIKGRYIQSHLLSRLEAIREKLKEQIKGVEAVQAKEVPLSWVGVAE
SVQVMGSFDGWSQGEHLSPEYTGSYMNFSATLFLRPGRYEIKFMVDDEWKLSPELPTG
EGLTKNNLLVVE

> Triticum aestivum W5GC57 (UniProtKB)

MECLTASFAARNACMEYKFVCPSKPACEKQWIPGRVLCYFTAYTNSSRRCKVATGVCP
VSPVVGRRSRWRSFAASLNLENGPAPSSSTSSSSGQASEQLTSAELKSLLADKERSKLLR
KLSEANQLNRFLKRQSQIKDDAIVKFRSELAVLELELQALVGLAEEIANFDVPSGSRKVN
GKYIQSHLLSRLEAVHDKVMVQIKDIESLRPREVAVHWVGMAENVQIMGSFDGWSHGE
AMSREYSGDYARFSATLRLRPGSYEIKFLVDGEWKLSSEYPITGEGLTQNNKLAVE

> Vitis vinifera XP 002275302
MNSLILQSSLQPALGCPENQVSWFSWKSTNLDWEDVRGLRCNVTRWNLRVGSQKCAIT
CQAFVGKQSMHQKLWRTYSMPINLDKESSSPFSQDYSSDNENASEDSPEELLDQPLGSD
ELKTLLVDSERTKLIKKLSEANQQNRILKRQLYIKEDALVNFKSELAVMELEVQALVSLA
EEIAKSGIPKGSRKINGKYIQSHLHSRLEAVHEKLKEQIKDVDAVQSKEVSIIWCGMAES
VQVMGTFDGWSQGEHLSPEYTGSFTKFSTTLMLRPGRYEIKFLVDGEWQLSPEFPTVGE
GLMENNLLIVK

> Zea mays PWZ22628
MECLTTSFPRNVGKEYICSSKLVSGNQWIPKRICCYVSCSTNSSRCHKFTDVACPMSPVI
RKRLRWRCFAASLNLEDGPAPSDSTSSSSEQTTDADGTTNGDVSENLLSRKPSSDELKAL
LADSERSRLLRRLSEANQYNRFLKRQVIFASRHLIKGKSTALSRNRVCKHEVLQIKDDAV
VKFKGELAVLELEMQALVGLAEEVANFDVPLGSRKINGKYIQSHLLSRLEAVHDKIMEQ
VKSVDALKTQEIPVYWIGMAENVQIMGSFDGWSQGEAMSMEYSGDYGRFSATLKLRP
GRYEIKFLVDGEWRLSPEYPTAGDGLTQNNILVVE

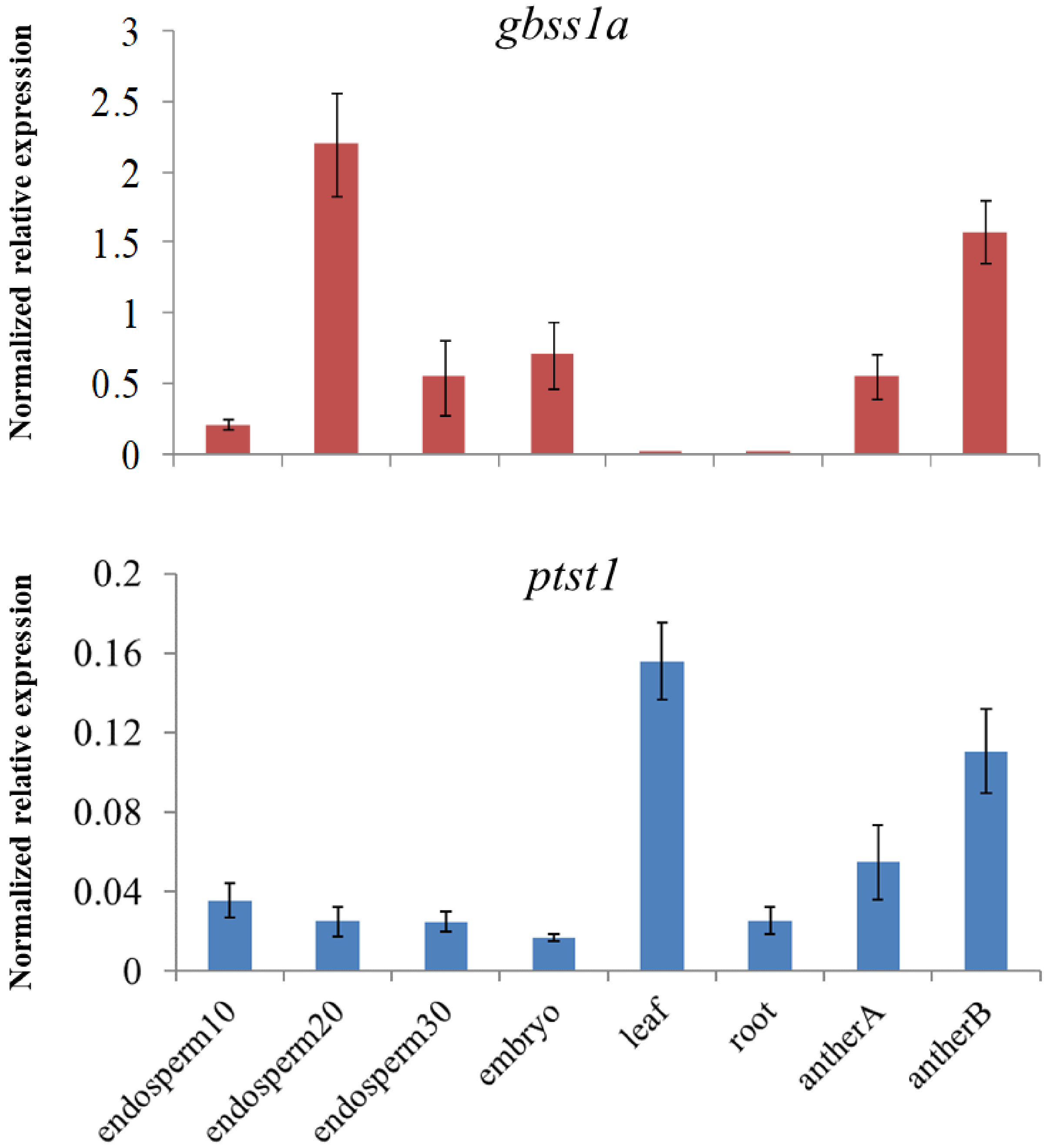

gbss1a
Normalized relative expression
3
2.5
2
1.5
1
0.5
0
ptst1
Normalized relative expression
0.2
0.16
0.12
0.08
0.04
0
endosperm10
endosperm20
endosperm30
embryo
leaf
root
antherA
antherB

Fig. S2

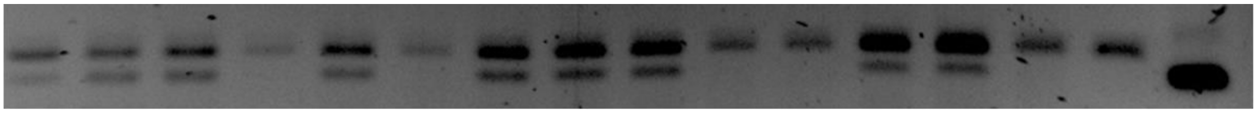

Fig. S3

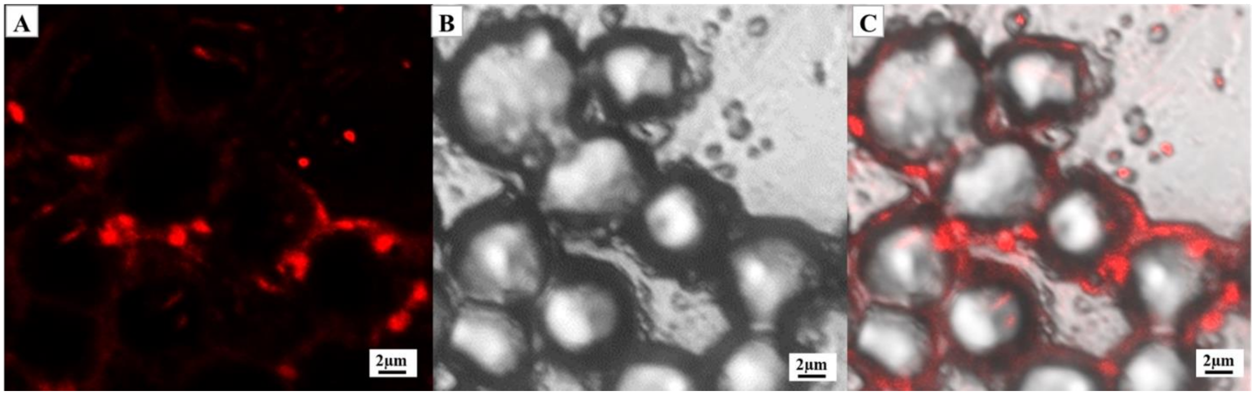

Fig. S4

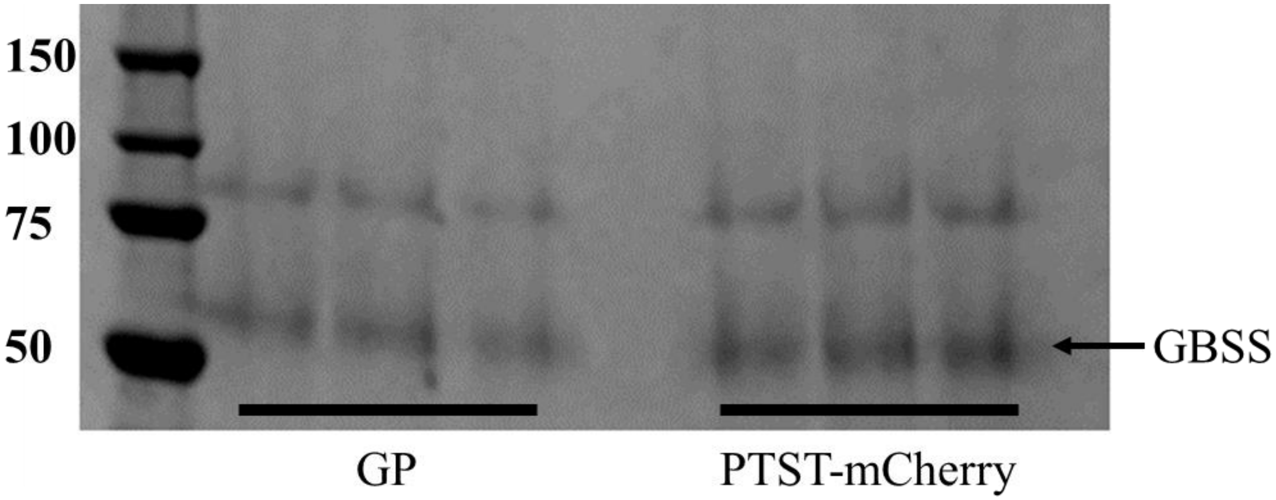

Fig. S5

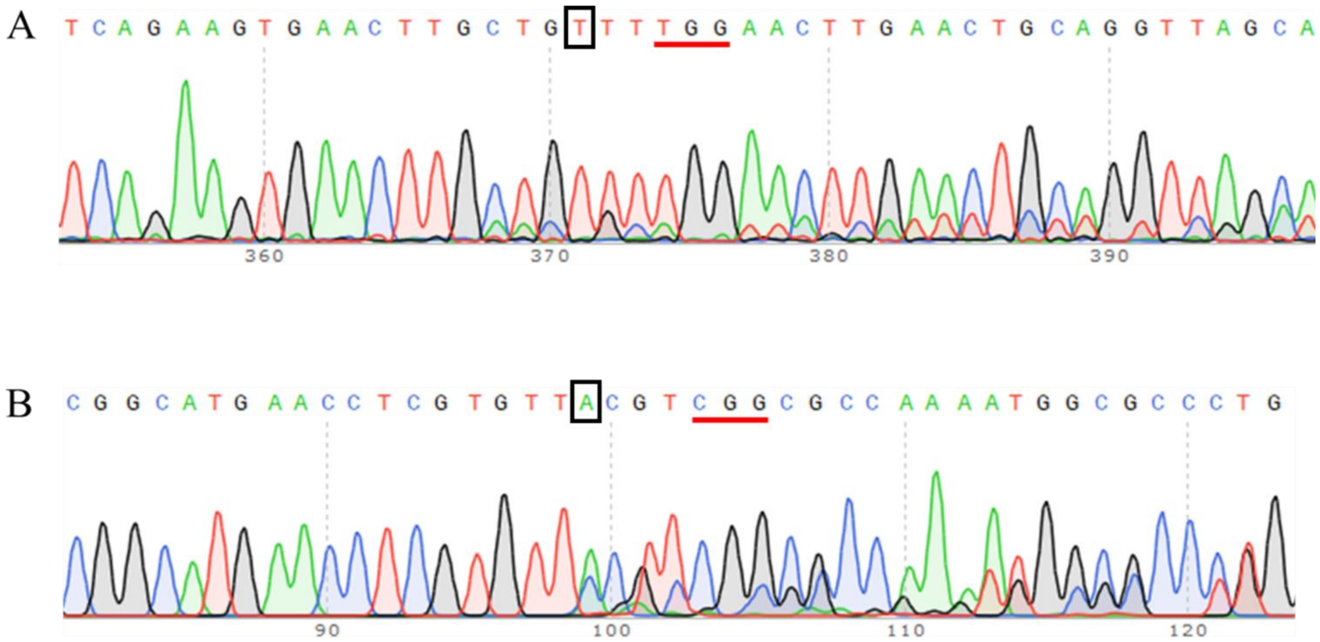
A
T C A G A A G T G A A C T T G C T G T T T T G G A A C T T G A A C T G C A G G T T A G C A
360
370
380
390
B
C G G C A T G A A C C T C G T G T T A C G T C G G C G C C A A A A T G G C G C C C T G
90
100
110
120

Fig. S6

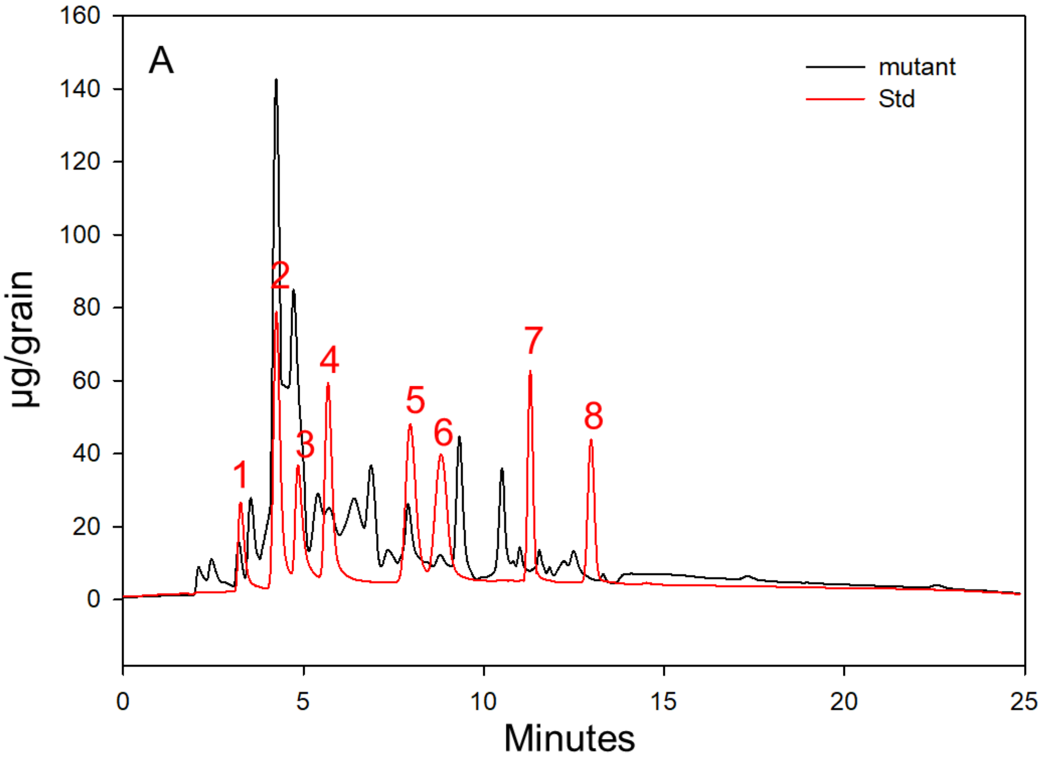
A
mutant
Std
1
2
3
4
5
6
7
8
160
140
120
100
80
60
40
20
0
µg/grain
0
5
10
15
20
25
Minutes

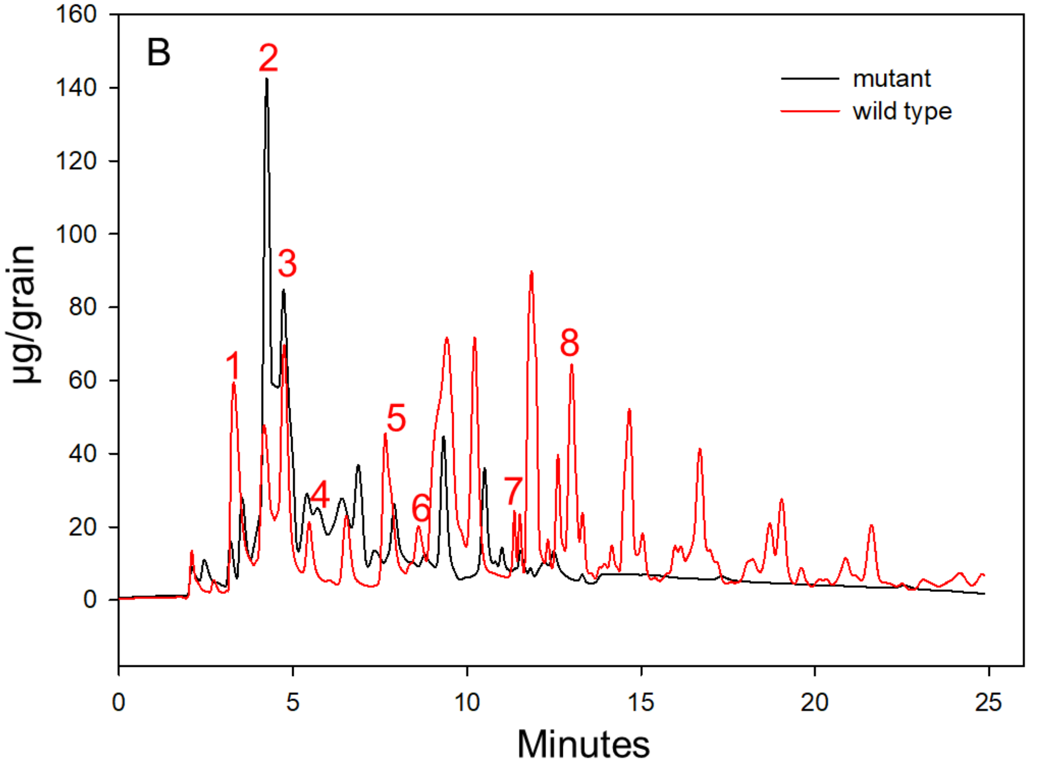
B
mutant
wild type
1
2
3
4
5
6
7
8
160
140
120
100
80
60
40
20
0
µg/grain
0
5
10
15
20
25
Minutes

Fig. S7

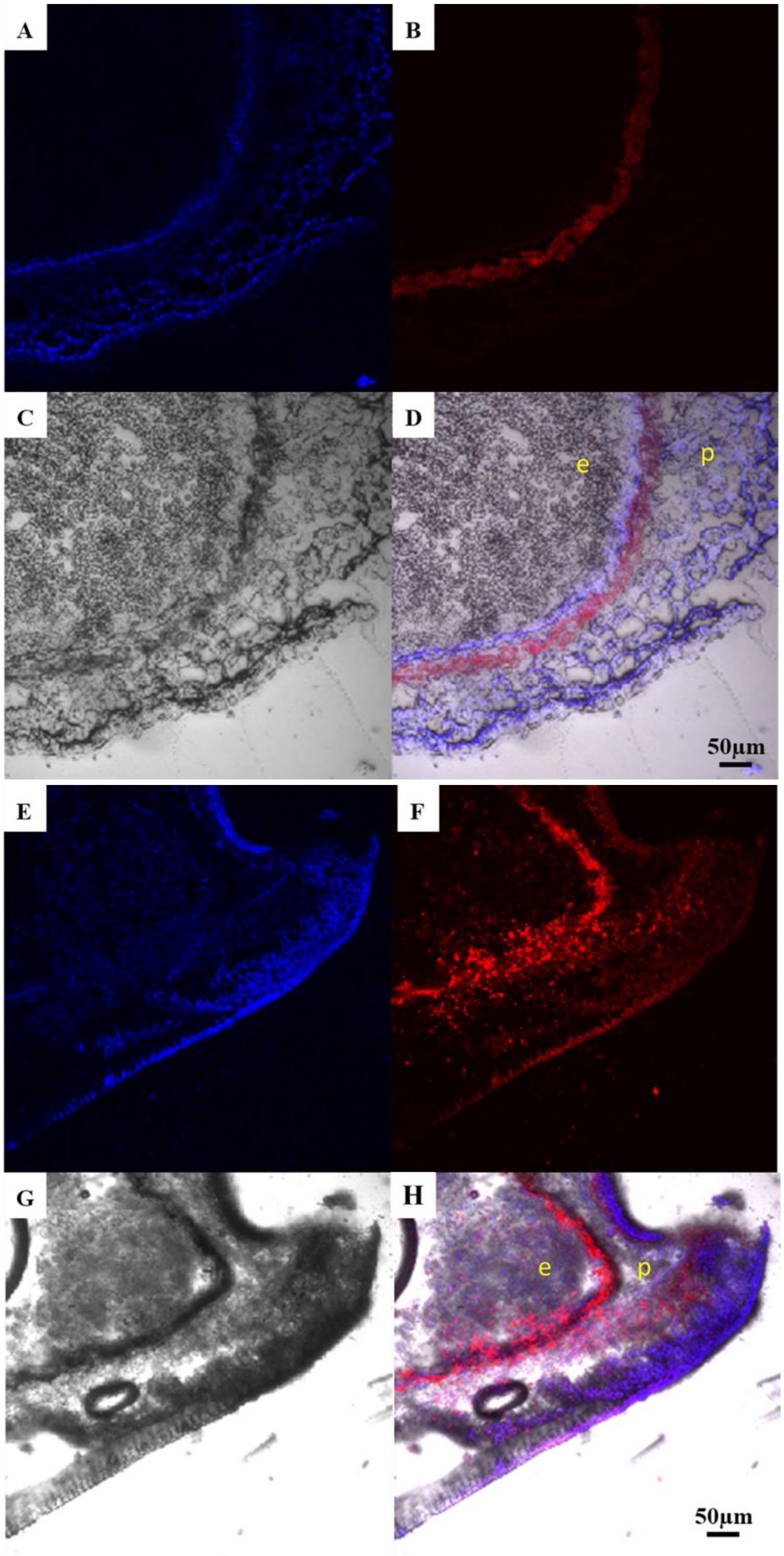

Fig. S8
